# Supplementary material for: Epilepsy-Induced High Affinity Blockade of the Cardiac Sodium Current INa by Lamotrigine; A Potential for Acquired Arrythmias
Source: Pharmaceuticals (Basel). 2022 Sep 29;15(10):1208. doi: 10.3390/ph15101208 (PMC9609666; doi:10.3390/ph15101208)
Supplement: Supplementary file 1 [file pharmaceuticals-15-01208-s001.zip › pharmaceuticals-1866350-supplementary.pdf]

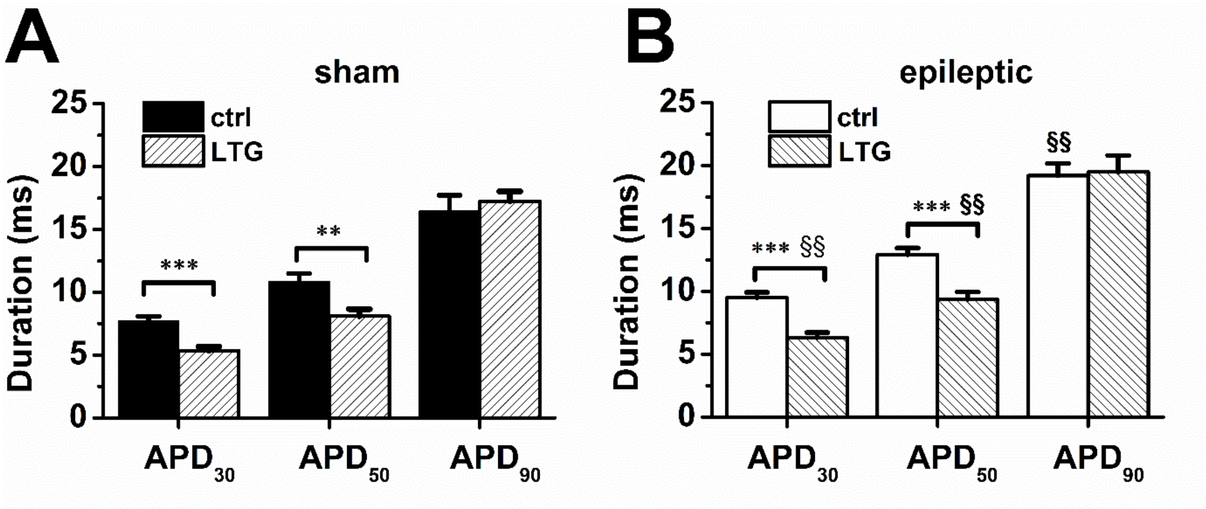

Figure S1. LTG decreased cardiac action potential duration (APD) at 30% and 50% of repolarization. (**A,B**) Action potential duration at 30% (APD_{30}), 50% (APD_{50}) and 90% (APD_{90}) of repolarization, from sham and epileptic rat cardiomyocytes in control conditions and after perfusion with LTG (1μmol/L). Bars indicate average value (± SEM). Tukey test using ANOVA one way. **indicates p<0.01 and ***indicates p < 0.001 (ctrl vs. LTG). §§ indicates p<0.01 (sham vs. epileptic). Number of animals: 3/group (ctrl and LTG). Number of cells: Sham (ctrl, LTG):21, 12. Epileptic (ctrl, LTG): 12, 16.

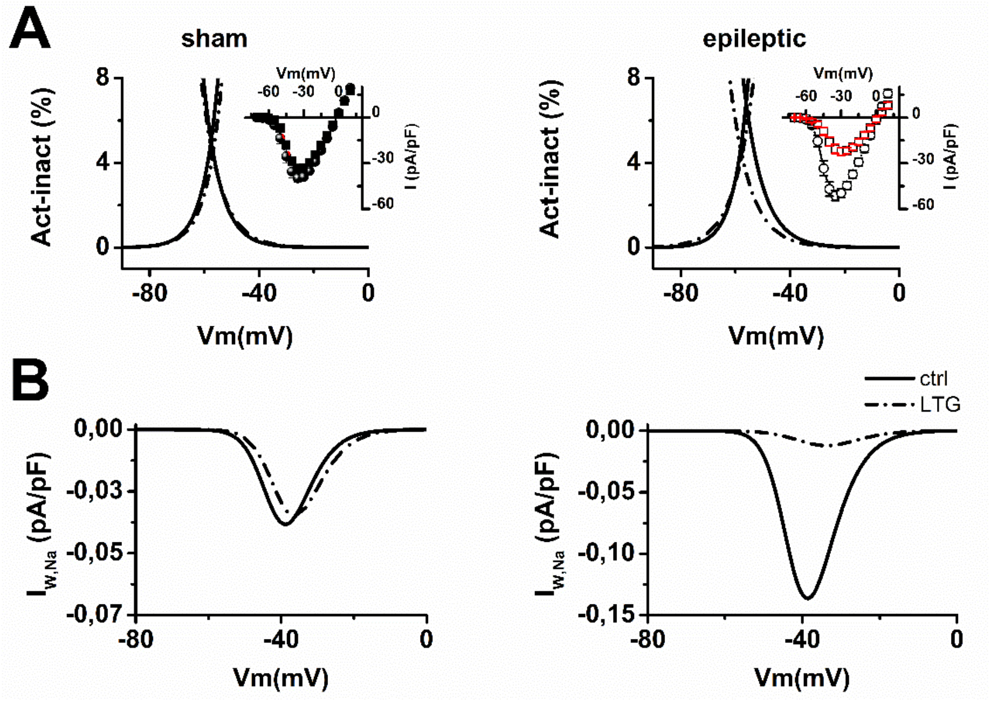

Figure S2. Sodium window current ($I_{W,Na}$) was flattened by LTG during epilepsy. (**A**) Activation-inactivation curves of I_{Na} for sham and epileptic in control (ctrl) and in presence of LTG (10μmol/L). Figure insets show the current-voltage relations comparison for control (circles) and LTG (squares). Number of cells: Sham (ctrl, LTG): 13, 14. Epilepsy (ctrl, LTG): 14, 13. (**B**) Window current calculated from the overlap of activation and inactivation curves showed in (A) and using the standard Hodgkin-Huxley formalism.
